# Testicular torsion in a catheterized geriatric 73-year-old patient, making an early diagnosis: a case report

**DOI:** 10.1093/jscr/rjab191

**Published:** 2021-05-18

**Authors:** Mahamudu A Ali, Mawuenyo Oyortey, Raymond S Maalman, Otchere Y Donkor, Henrietta Quarshie

**Affiliations:** Department of Surgery, School of Medicine, University of Health and Allied Sciences, Ho, Ghana; Department of Basic Medical Sciences, School of Medicine, University of Health and Allied Sciences, Ho, Ghana; Department of Surgery, School of Medicine, University of Health and Allied Sciences, Ho, Ghana; Department of Basic Medical Sciences, School of Medicine, University of Health and Allied Sciences, Ho, Ghana; Department of Basic Medical Sciences, School of Medicine, University of Health and Allied Sciences, Ho, Ghana; School of Medicine, University of Health and Allied Sciences, Ho, Ghana

## Abstract

Testicular torsion is a urologic emergency that requires surgical intervention. Its diagnosis is rarely made in elderly men especially the subset of men on urethral catheter. As a result, delayed diagnosis and surgical exploration occur leading to testicular infarction with necrosis, abscess formation and ultimately orchidectomy. We report a 73-year-old urologic patient referred with a 2-month history of transurethral catheterization to relieve retention of urine with subsequent scrotal pains and fever. Physical examination showed left hemi-scrotal swelling and normal right hemi-scrotal findings. A Doppler scan done showed an intratesticular fluid collection with no blood flow in the left testes. This case illustrates the need to include testicular torsion when diagnosing geriatric men with transurethral catheter presenting with any acute scrotal pains. We, therefore, recommend a detailed history and physical examination in addition to a colour Doppler ultrasound scan in making a diagnosis.

## INTRODUCTION

One of the rarely seen urological emergencies in the adult and elderly male population is spermatic cord torsion (SCT) with fewer than 10 cases reported in literature among urological patients aged 70 years and above [1, 2]. SCT occurs when there is twisting of the spermatic cord to various degrees leading to the cessation of intratesticular perfusion and resultant infarction, necrosis, severe pains and sometimes abscess formation [1, 3]. The extent of testicular rotation has also been directly correlated with the time to testicular necrosis and, therefore, the probability of salvage [3]. As the salvage rate of an affected testis is highly dependent on the duration and degree of torsion, a high index of suspicion and detailed history about the onset is required from practitioners in making an early diagnosis [4, 5].

A decrease in intratesticular perfusion starts with obstruction of venous outflow when the degree of spermatic cord twist is <360° and cessation of arterial flow when the testis twists 360° or greater, leading to the testicular ischaemia [6]. As time passes, the testis becomes enlarged and a complete absence of blood flow is observed by Doppler examination [6]. Early and timely diagnosis and surgical intervention may salvage the affected testes; however, if there is an excessive delay in patient presentation, this approach will result in orchidectomy [6–8]. Testicular torsion is diagnosed based on a careful physical examination and appropriate colour Doppler ultrasound (CDU) [9].

Unfortunately, the early diagnosis of testicular torsion may be missed, if it occurs in a rare situation such as in geriatric patients or conditions such as a patient with an indwelling urethral catheter. Complications due to chronic indwelling urethral catheter such as infectious–cystitis, prostatitis, epididymitis, urethritis and periurethral abscess [10] may be misleading to the prescribers. This case report is therefore to create awareness among clinicians for early diagnosis of testicular torsion in geriatric male patients with an indwelling urethral catheter.

## CASE REPORT

We present a 73-year-old man who was referred to a hospital after 9 days of management as acute epididymo-orchitis by a primary caregiver.

He presented with sudden onset of left hemi-scrotal pains. This was his first episode of experiencing such pains that were so severe that he could not continue with his usual activities. He had been very well after a transurethral catheterization 2 months prior, on account of acute retention of urine from clinically suspected benign prostatic hyperplasia and had since been regularly changing the catheter. There was no prior history of any urethritis, urethral discharges, scrotum swelling, fever, chills, rigours, a feeling of malaise, haematuria, lower urinary tract discomfort, suprapubic pains or trauma. He had no co-morbidities like diabetes. He was empirically treated with oral ciprofloxacin and analgesia and discharged. Even though he noticed an improvement in the pains, he developed a progressively worsening left hemi-scrotal swelling associated with generalized malaise.

**Figure 1:**
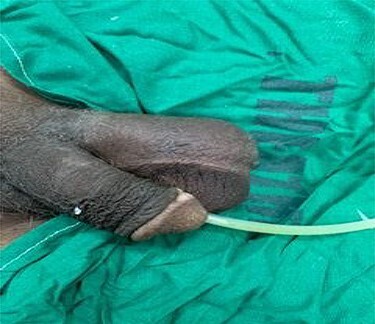
Swollen left hemi-scrotum. A urethral catheter *in situ.*

Physical examination on the patient revealed a swollen left hemi-scrotum (Fig. 1) with differential warmth but non-tender. The testis, epididymis and ipsilateral spermatic cord felt indurated on palpation. The contralateral testicle and epididymis appeared normal. The prostate felt benign and non-tender on digital rectal examination.

**Figure 2:**
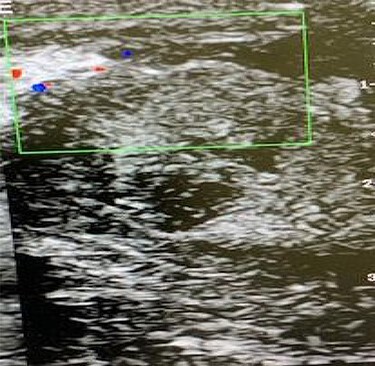
CDU scan showing lack of intratesticular blood flow and a collection of fluid within the testicular parenchyma.

A CDU scan was done, and it showed an intratesticular fluid collection with no blood flow into the left testis and normal right hemi-scrotal findings (Fig. 2).

**Figure 3:**
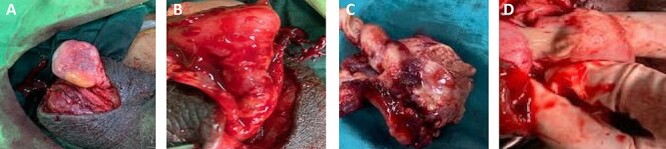
(**A**) A left dark testicle and a normal right testis that was relatively easy to exteriorise from the scrotal sac; (**B**) intravaginal spermatic cord twisting with copious exudate within the tunica vaginalis; (**C**) specimen after orchidectomy with about 540 degrees twist and (**D**) inadvertent rupture showing intratesticular pus.

Other investigations showed mild leucocytosis but no pus cells in the urine. He had scrotal exploration done, and the findings were an intravaginal torsion, oedematous left scrotum with a swollen and necrotic testis and a normal right testicle (Fig. 3a). There was copious exudate within the tunica vaginalis (Fig. 3b). He had left orchidectomy and right orchidopexy done during which a 540-degree twisting of the left spermatic cord was found (Fig. 3c). The necrotic testes had an intratesticular abscess (Fig. 3d). The histopathological examination confirmed infarction without signs of testicular tumour. His condition improved and he was satisfied with the management. No adverse and anticipated events afterwards detected.

## DISCUSSION

Testicular torsion is much common among adolescents and neonates with few reported cases in the geriatric population [2, 11]. There are far fewer cases reported in men over 70 years old, and none have been reported with a transurethral catheter *in situ*.

Most scrotal pain in the elderly is often attributed to infective causes such as epididymitis or orchitis especially in inpatient with transurethral catheters *in situ* [10]. Other common differentials made in a person presenting with scrotal pains include: testicular tumour, hernia, infected hydrocele and varicocele. This affirms the rare occurrence of testicular torsion in the elderly [2, 12] with no reported case in the subpopulation of urological cases with transurethral catheters and probably the first reported case in Ghana.

The majority of other pathologies are diagnosed clinically through history and physical examination. However, delayed diagnosis is noted among elderly men reporting with testicular torsion and very often this is through Doppler ultrasound scan [13, 14]. Our case who presented with the classical sudden onset of unilateral scrotal pains was eventually diagnosed by means of a CDU scan. This may be attributed to low index of suspicion in this age group because of pathologies and related procedures, which are noted risk factors for the development of orchitis and epididymitis. Even though Doppler imaging in our case correctly diagnosed SCT, it did not translate to a better outcome in terms of testicular viability or salvage and the patient eventually had an orchidectomy as the mainstay of treatment. Does the near 100% sensitivity and specificity of Doppler ultrasound in the diagnosis of testicular torsion reflect in testes salvage rate? The patient and relatives were informed about the way forward and management. They agreed and gave their consent to that effect.

## CONCLUSION

The management of this case illustrates how a low index of suspicion continues to delay the diagnosis of testicular torsion and impact poor testes salvage despite classical presentations. Testicular torsion must be included as a differential diagnosis in the evaluation of men with transurethral catheters presenting with acute scrotal pain. We recommend a detailed history and physical examination in cases of this nature.

## Data Availability

The materials used for the current study are available with the corresponding author on reasonable request.
